# Parental smoking and young adult offspring psychosis, depression and anxiety disorders and substance use disorder

**DOI:** 10.1093/eurpub/ckac004

**Published:** 2022-01-29

**Authors:** Marian Sarala, Antti Mustonen, Anni-Emilia Alakokkare, Caroline Salom, Jouko Miettunen, Solja Niemelä

**Affiliations:** 1 Research Unit of Clinical Neuroscience, University of Oulu, Oulu, Finland; 2 Faculty of Medicine and Health Technology, University Consortium of Seinäjoki, Tampere University, Tampere, Finland; 3 Medical Research Center Oulu, Oulu University Hospital and University of Oulu, Oulu, Finland; 4 Institute for Social Science Research, University of Queensland, Queensland, Australia; 5 Center for Life Course Health Research, University of Oulu, Oulu, Finland; 6 Addiction Psychiatry Unit, Department of Psychiatry, Turku University Hospital, Turku, Finland; 7 Department of Psychiatry, University of Turku, Turku, Finland

## Abstract

**Background:**

To study the associations between maternal smoking during pregnancy and paternal smoking before pregnancy and adult offspring psychiatric disorders.

**Methods:**

Prospective general population cohort study in Northern Finland, with people from the Northern Finland Birth Cohort 1986: 7259 subjects (77% of the original sample). Data on parental smoking were collected from parents during pregnancy using questionnaires. Outcomes were offspring’s register-based diagnoses: any psychiatric disorder, any non-organic psychosis, mood disorder, anxiety disorder and substance use disorder (SUD) until the age of 29–30 years. Maternal smoking during pregnancy and paternal smoking before pregnancy were pooled to three-class variables: (i) none; (ii) 1–9 and (iii) ≥10 cigarettes/day. Information regarding both parents’ alcohol use during pregnancy and at offspring age 15–16 years, maternal education level, family structure, parental psychiatric diagnoses and offspring gender, smoking, intoxication frequency and illicit substance use at the age of 15–16 years were investigated as covariates.

**Results:**

In the multivariable analyses, maternal smoking during pregnancy did not associate with the studied outcomes after adjusting for offspring smoking and other substance use at offspring age 15–16 years and parental psychiatric disorders. However, paternal smoking ≥10 cigarettes/day before pregnancy [hazard ratio (HR) = 5.5, 95% confidence interval (CI) 2.7–11.2, *P *<* *0.001] and paternal psychiatric disorders (HR = 1.7, 95% CI 1.1–2.8, *P *=* *0.028) associated with offspring SUD after adjustments.

**Conclusions:**

Information across the offspring life course is essential in exploring the association between parental smoking and offspring psychiatric disorders. Paternal smoking before pregnancy and paternal psychiatric disorders may act as modifiers in elevating the risk of substance-use-related problems among offspring.

## Introduction

Maternal smoking during pregnancy is a common risk factor for offspring health problems. In European countries, ∼4–19% of women smoke during pregnancy.^1^ Prenatal smoking may cause epigenetic modifications as well as changes in the structure and function of the fetal brain.^2^ This may represent risks for psychiatric morbidity later in life.^3^^,^^4^ In a comprehensive population-based register-linkage study, prenatal smoking exposure was found to associate with psychiatric diagnoses in 18-year-old offspring, including mood, anxiety and substance use disorders (SUDs) and psychotic disorders.^5^ However, in this population-based study, the child-related confounding factors assessed were connected only to the child’s birth (i.e. gestational age, birth weight and Apgar score) and not to later developmental stages, which may also have an influence on offspring psychiatric morbidity in early adulthood. Indeed, prenatal smoking has been shown to predict offspring smoking, alcohol and other drug use in adolescence,^6^^,^^7^ which has further been associated with an increased risk of anxiety, depression and SUDs in young adulthood.^8–11^ There are inconsistent findings for the association of prenatal smoking with offspring psychiatric disorders, which are mainly caused by different confounding factors assessed in the studies. Studies that examined the confounding factors related to the child’s birth more often reported associations between prenatal smoking and offspring psychiatric disorders than studies that considered also parental psychiatric disorders or offspring behavior at later developmental stages.^12^

In addition to maternal smoking, paternal smoking before pregnancy may also have an impact on offspring health. Worldwide ∼23% of the men at reproductive age smoke tobacco.^13^ Smoking influences sperm by creating epigenetic markers, increasing oxidative damage to sperm DNA, and changing extracellular signals. These factors may alter the molecular composition of the fertilized embryo and subsequently induce a range of physical health impacts in offspring.^14^^,^^15^ Regarding mental health, although one birth cohort study showed no effect of paternal smoking during pregnancy on 22- to 23-year-old offspring hypomania outcomes,^16^ a recent review implicates paternal smoking in offspring Attention-Deficit/Hyperactivity Disorder (ADHD).^17^ Nonetheless, research into the association between paternal smoking before pregnancy and offspring psychiatric disorders in early adulthood is sparse, and models, which account for a range of potentially confounding factors, including maternal smoking, are rare.^17^

We aimed to study the associations of maternal smoking during pregnancy and paternal smoking before pregnancy with offspring psychiatric disorders at the age of 29–30 years using the Northern Finland Birth Cohort (NFBC) 1986. We hypothesize that the associations between maternal smoking during pregnancy and offspring’s subsequent psychiatric disorders attenuate after confounding factors over the life course have been considered. Therefore, we also consider parental and offspring factors during pre-pregnancy and gestation, at birth and during adolescence.

## Methods

### Participants

The associations between maternal smoking during pregnancy and adult offspring mental health were studied in the NFBC 1986. NFBC includes 99% of all births from the two northernmost provinces in Finland, incorporating all live born children (*n* = 9432) with an expected birthday between 1 July 1985 and 30 June 1986. Data were collected from the parents during pregnancy (*n* = 8986, 95.3% for mothers and *n* = 8095, 85.8% for fathers) using questionnaires and from the parents and the child at offspring age 15–16 years (*n* = 7586, 80.4% for consenting offspring participants). Offspring who had psychiatric disorders before age 16 years were excluded. Since the association between maternal smoking during pregnancy and adolescent drug use via externalizing behavior has already been reported in the NFBC86,^18^ we focused on the less studied diagnoses and adolescence onset illnesses. In all, 7259 children (49.5% boys) participated in the study in 2001–02, when the study members were aged 15–16 years (Supplementary data 1). For more information about the study design, see NFBC data collection web page.^19^ Data on psychiatric diagnoses were cumulatively collected from the four national registries from the participants’ age 15–16 years through age 29–30 years, i.e. until the end of 2016. The study was approved by the Ethical committee of the Northern Ostrobothnia Hospital District (statement number 108/2017).

### Exposures

Data on maternal self-reported smoking during pregnancy were gathered from a questionnaire issued to the mother at her first visit to the maternal healthcare center (approximately 10–12th gestational week). The questionnaire was to be returned by the 24th week. Mothers were asked: ‘Did you smoke after two months of pregnancy?’ (yes/no) and ‘How many cigarettes did you smoke in a day?’ Answers to the latter question were pooled into three classes: (i) none; (ii) 1–9; and (iii) ≥10 cigarettes.

Data on paternal smoking were acquired from a questionnaire issued to the fathers during pregnancy. Fathers were asked: ‘How many cigarettes do you smoke in a day?’ and answers were pooled to a three-class variable: (i) none; (ii) 1–9; and (iii) ≥10 cigarettes/day. Fathers were also asked ‘How many years have you smoked?’, which was used to determine whether they had smoked before pregnancy (yes/no).

### Outcomes

Information on offspring diagnosed psychiatric disorders was collected from nationwide registers: The Care Register for Health Care 2001–2015 and the Register of Primary Health Care Visits 2001–2015 of the National Institute for Health and Welfare, disability pensions of the Finnish Center for Pensions 2001–2016 and the medication reimbursement register of the Social Insurance Institution of Finland 2001–2015. The Care Register contains data on patients discharged from inpatient care, and since 1998 also data on specialized outpatient care. The Register of Primary Health Care Visits covers all outpatient primary health care delivered in Finland. For more information about the registers, see^20^ Supplementary data 1. Five outcome variables were constructed from the psychiatric disorders. The first variable (any psychiatric disorder) included all the studied psychiatric disorders. The other outcome groupings were any non-organic psychosis, anxiety disorder, mood disorder and any SUD (Supplementary data 2). A cohort member could belong to several diagnostic classes. The reference group in all variables was those participants without that diagnosis. The SUD group comprised diagnoses of dependence and harmful use of alcohol and/or other drugs, including nicotine.

### Covariates

Potential confounding factors were included according to reports in the literature. Maternal and paternal self-reported use of any alcohol (yes/no) was collected during pregnancy and at offspring age 15–16 years.

Information on education level of both parents was gathered from the 15- to 16-year questionnaire issued to the families. The parental education level was divided into two groups: (i) having completed high school, i.e. schooling for 12 years and (ii) not completing high school, i.e. <12 years of schooling.

Information on family structure was collected when the cohort member was aged 15–16 years. Families were classified as (i) both parents living with the subject all the time and (ii) other families.

Low birth weight (<2500 g) and low Apgar score at 15 min (<7 points) were considered as potential confounding factors.

Data on maternal and paternal psychiatric diagnoses were based on parental diagnoses recorded in the nationwide registers. These registers include: (i) Register of Health Care during the years 1969–2016 (Hospital Discharge Register until 1994). This register also includes visits to specialized outpatient health care since 1998; (ii) disability pensions of the Finnish Center for Pensions (1965–2016) and (iii) The Register of Primary Health Care Visits (2011–2016).

Information on illicit drug use by offspring at the age of 15–16 years was collected with two self-reported questions: ‘Have you used marihuana or hashish?’ and ‘Have you used ecstasy, heroin, cocaine, amphetamine, LSD or other similar intoxicating drugs?’ These questions were combined into a binary variable: other drug use (no/yes). A person was categorized as yes if she/he had used any of the above-mentioned drugs at least once by the age of 15–16 years.

Offspring daily smoking at the age of 15–16 years was self-reported in the questionnaire. Study members were asked: ‘Do you smoke now?’ with options: (i) not at all; (ii) occasionally; (iii) once a week; (iv) on 2–4; (v) 5–6; and (vi) 7 days a week. A three-class variable was formed: (i) no smoking; (ii) occasional smoking (options 2–5); and (iii) daily smoking.

Intoxication frequency at offspring age 15–16 years was also self-reported: ‘How many times have you been drunk during the past 30 days?’ The options were: Never, 1–2, 3–5, 6–9, 10–19, 20–39 or 40 times or more, and this was categorized as a two-class variable of being intoxicated (i) 0–2 times and (ii) ≥3 times during the last 30 days.

### Analytical methods

Cross-tabulation and Chi-square tests were used to determine significant associations of potential confounders with offspring psychiatric diagnoses. Cox regression analyses were conducted to examine the associations of (i) maternal smoking during pregnancy and (ii) paternal smoking pre-pregnancy with each offspring psychiatric outcome to yield hazard ratios (HRs) and their 95% confidence intervals (95% CIs). The reference groups were the non-smoking mothers during pregnancy and the non-smoking fathers before pregnancy, respectively. Where significant associations were found, we adjusted for covariates identified as significant in Chi-squared analyses above. We adjusted the maternal smoking analyses with maternal alcohol use during pregnancy, gender, maternal education level, family structure, parental psychiatric diagnoses, paternal smoking and offspring’s alcohol intoxication frequency, daily smoking and other drug use by the age of 15–16 years. For paternal smoking before pregnancy analyses, the adjustments were the same, except in the case of offspring SUD in adulthood, where we also included paternal alcohol use during pregnancy and parental alcohol use at offspring age 15–16 years. An additional model including both maternal smoking during pregnancy and paternal smoking before pregnancy was performed in a similar manner. As a *post* *hoc* analysis, we also examined potential interaction between paternal daily smoking before pregnancy (yes/no) and any paternal psychiatric disorder in relation to SUD in offspring. Analyses were adjusted as above including also paternal alcohol use during pregnancy. Statistical interactions were studied under multiplicative models using HR. The studied interactions were maternal smoking during pregnancy or paternal smoking before pregnancy × offspring intoxication frequency, daily smoking or other drug use at the age of 15–16 years × offspring any psychiatric diagnosis, mood disorder, anxiety disorder or SUD and paternal smoking before pregnancy × any paternal psychiatric diagnosis × offspring SUD. The results were considered statistically significant at level *P *<* *0.05. All statistical tests were two-tailed and performed with SPSS software (version 24).

## Results

At offspring age 29–30 years, 14.5% of males and 21.9% of females had any psychiatric disorder (Table 1). Nearly one in five of participating offspring (18.6%) had been exposed to maternal smoking during pregnancy; half of these (9.5% of the sample) to >10 cigarettes per day. Over a third (35.5%) had a father who had smoked before pregnancy and 30.2% of those fathers smoked ≥10 cigarettes daily (data not shown). At offspring age 15–16 years, 26.8% of those who smoked daily and 30.2% of those reporting illicit drug uses had a recorded psychiatric disorder. Moreover, of the 29- to 30-year-old offspring with any psychiatric disorder, 25.7% had a mother and 26% had a father with a diagnosed psychiatric disorder. Low birth weight (<2500 g) and Apgar scores (<7 points at 15 min) were not associated with offspring psychiatric disorders (Table 1).

**Table 1:** Offspring and parental characteristics and offspring psychiatric diagnoses at the age of 29–30 years

	Total *n* = 7259		No psychiatric disorder		Any psychiatric disorder		Anxiety disorder		Mood disorder		SUD		Any psychosis	
Characteristics	*N*	%	*n*	%	*n*	%	*n*	%	*n*	%	*n*	%	*n*	%
Gender	7259	100												
Male	3593	49.5	3072	85.5	521	14.5	240	6.7	207	5.8	105	2.9	72	2.0
Female	3666		2862	78.1	804	21.9	432	11.8	397	10.8	63	1.7	55	1.5
Maternal smoking during pregnancy	7259	100												
No			4868	82.4	1040	17.6	511	8.6	160	8.0	120	2.0	106	1.8
1–9 cigarettes a day			521	79.1	138	20.9	78	11.8	347	8.2	22	3.3	10	1.5
≥10 cigarettes a day			545	78.8	147	21.2	83	12.0	236	10.8	26	3.8	11	1.6
Paternal smoking before pregnancy	6331	87.2												
No			3370	82.5	713	17.5	362	8.9	335	8.2	62	1.5	72	1.8
1–9 cigarettes a day			273	82.0	60	18.0	25	7.5	24	7.2	12	3.6	3	0.9
≥10 cigarettes a day			1534	80.1	381	19.9	201	10.5	170	8.9	67	3.5	31	1.6
Maternal alcohol use during pregnancy	7172	98.8												
No			5144	82.1	1123	17.9	568	9.1	503	8.0	136	2.2	111	1.8
Yes			719	79.4	186	20.6	94	10.4	92	10.2	28	3.1	14	1.5
Maternal education, years	6166	84.9												
≥12			1682	83.3	337	16.7	158	7.8	154	7.6	32	1.6	38	1.9
<12			3379	81.5	768	18.5	394	9.5	341	8.2	97	2.3	69	1.7
Family type	6198	85.4												
Both parents			4064	83.8	787	16.2	383	7.9	342	7.1	76	1.6	81	1.7
One parent or other			1021	75.8	326	24.2	178	13.2	162	12.0	55	4.1	28	2.1
Apgar score at 15 min	4846	66.8												
<7			5	71.4	2	28.6	0	0.0	1	14.3	0	0.0	0	0.0
≥7			3967	82.0	872	18.0	433	8.9	415	8.6	116	2.4	83	1.7
Birth weight (g)	7259	100												
<2500			184	78.3	51	21.7	23	9.8	24	10.2	8	3.4	6	2.6
≥2500			5750	81.9	1274	18.1	649	9.2	580	8.3	160	2.3	121	1.7
Intoxication frequency at ages 15–16 years^a^	6070	83.6												
0–2 times			4556	83.0	935	17.0	459	8.4	408	7.4	99	1.8	91	1.7
Three times or more			441	76.2	138	23.8	69	11.9	74	12.8	33	5.7	15	2.6
Daily smoking at the age of 15–16 years	6526	89.9												
No			4719	83.0	965	17.0	494	8.7	431	7.6	82	1.4	93	1.6
Yes			616	73.2	226	26.8	116	13.8	117	13.9	60	7.1	20	2.4
Illicit drug use at the age of 15–16 years	6222	85.7												
No			4783	83.4	949	16.6	476	8.3	411	7.2	99	1.7	85	1.5
Yes			342	69.8	148	30.2	68	13.9	80	16.3	36	7.3	23	4.7
Maternal psychiatric disorder^b^	7259	100												
No			4876	83.6	959	16.4	475	8.1	425	7.3	110	1.9	98	1.7
Yes			1058	74.3	366	25.7	197	13.8	179	12.6	58	4.1	29	2.0
Paternal psychiatric disorder^b^	7259	100												
No			4923	83.6	969	16.4	484	8.2	434	7.4	102	1.7	85	1.4
Yes			1011	74.0	356	26.0	188	13.8	170	12.4	66	4.8	42	3.1

SD, Standard deviation.

a Past 30 days.

b Recorded at offspring age 15–16 years.

In unadjusted Cox regression analyses, the risk of any psychiatric disorder was increased in offspring whose mothers smoked 1–9 cigarettes (HR = 1.2, 95% CI 1.02–1.4) and ≥10 cigarettes daily (HR = 1.3, 95% CI 1.05–1.5) during pregnancy (Table 2). The risk for offspring mood disorder was higher if the mother smoked ≥10 cigarettes per day (HR = 1.4, 95% CI 1.1–1.8). The risk of anxiety disorder was elevated in offspring whose mothers smoked 1–9 cigarettes (HR = 1.4, 95% CI 1.1–1.8) and ≥10 cigarettes daily (HR = 1.4, 95% CI 1.1–1.8). The risk for offspring whose mothers smoked during pregnancy was highest for SUD (HR = 1.6, 95% CI 1.05–2.6 for 1–9 cigarettes and HR = 1.9, 95% CI 1.2–2.8 for ≥10 cigarettes daily). Maternal smoking during pregnancy did not increase the risk of offspring psychotic disorder at the age of 29–30 years. The associations between maternal smoking during pregnancy and all the studied adult offspring psychiatric outcomes became non-significant after adjusting for gender, family structure, parental psychiatric disorder and offspring illicit drug use at the age of 15–16 years (Table 3). The risk of SUD also remained associated with frequency of offspring alcohol intoxication during the last 30 days and daily smoking at the age of 15–16, but not with maternal smoking during pregnancy. In additional multivariable models (data not shown) that included both maternal smoking during pregnancy and paternal smoking before pregnancy and aforementioned confounding factors, no statistically significant associations between parental smoking variables and any outcomes under study were observed.

**Table 2:** Unadjusted Cox regression analyses for offspring psychiatric diagnoses with parental smoking

		*N*		1–9 cigarettes	≥10 cigarettes
Characteristics	Events	Censored	Total	HR (95% CI)	HR (95% CI)
Maternal smoking during pregnancy					
Any psychiatric diagnosis	1325	5934	7259	1.2 (1.02–1.4)	1.3 (1.05–1.5)
Mood disorder	604	6655	7259	1.02 (0.8–1.4)	1.4 (1.1–1.8)
Anxiety disorder	672	6587	7259	1.4 (1.1–1.8)	1.4 (1.1–1.8)
Psychotic disorder	127	7132	7259	0.8 (0.4–1.6)	0.9 (0.5–1.6)
SUD	168	7091	7259	1.6 (1.05–2.6)	1.9 (1.2–2.8)
Paternal smoking before pregnancy					
Any psychiatric diagnosis	1154	5177	6331	1.1 (0.8–1.4)	1.2 (1.03–1.3)
Mood disorder	529	5802	6331	0.9 (0.6–1.3)	1.1 (0.9–1.3)
Anxiety disorder	588	5743	6331	0.8 (0.6–1.3)	1.2 (1.01–1.4)
Psychotic disorder	106	6225	6331	0.5 (0.2–1.6)	0.9 (0.6–1.4)
SUD	141	6190	6331	2.4 (1.3–4.5)	2.3 (1.6–3.3)

**Table 3:** Adjusted Cox regression analyses for offspring psychiatric diagnoses with maternal smoking during pregnancy

	Any psychiatric disorder	Mood disorder	Anxiety disorder	SUD
Characteristics	HR (95%CI)	HR (95% CI)	HR (95% CI)	HR (95% CI)
Maternal smoking during pregnancy				
<9 cigarettes	1.05 (0.8–1.3)	0.8 (0.5–1.1)	1.2 (0.8–1.6)	1.0 (0.5–1.9)
≥10 cigarettes	1.06 (0.8–1.3)	1.0 (0.7–1.3)	1.1 (0.8–1.6)	0.9 (0.5–1.8)
Maternal alcohol use during pregnancy (yes)	1.02 (0.8–1.2)	1.2 (0.9–1.5)	1.0 (0.7–1.3)	1.2 (0.7–2.1)
Female gender	1.6 (1.3–1.8)	1.7 (1.4–2.2)	1.9 (1.5–2.3)	0.5 (0.3–0.7)
Maternal education <12 years	1.05 (0.9–1.2)	1.0 (0.8–1.2)	1.2 (0.9–1.5)	0.9 (0.6–1.5)
Family structure				
One parent or other	1.2 (1.05–1.4)	1.3 (1.1–1.7)	1.3 (1.01–1.6)	1.7 (1.1–2.6)
Maternal psychiatric diagnosis (yes)	1.6 (1.4–1.8)	1.9 (1.5–2.3)	1.5 (1.2–1.9)	1.9 (1.2–2.9)
Paternal psychiatric diagnosis (yes)	1.7 (1.4–2.0)	1.6 (1.3–2.0)	1.6 (1.3–2.0)	2.9 (1.9–4.5)
Offspring daily smoking at the age of 15–16 years (yes)	1.05 (0.9–1.3)	1.2 (0.9–1.6)	1.05 (0.8–1.4)	2.6 (1.6–4.4)
Offspring intoxication frequency at the age of 15–16 years, past 30 days (yes)	1.2 (0.96–1.5)	1.2 (0.9–1.7)	1.1 (0.8–1.5)	1.7 (1.01–2.9)
Offspring other drug use at age of 15–16 years (yes)	1.9 (1.6–2.4)	2.2 (1.7–3.0)	1.6 (1.2–2.2)	2.4 (1.4–4.0)

The unadjusted analyses showed increased risk of any psychiatric disorder (HR = 1.2, 95% CI 1.03–1.3) and anxiety disorder (HR = 1.2, 95% CI 1.01–1.4) for those offspring whose fathers smoked 1–9 cigarettes daily before pregnancy (Table 2). As for mothers’ smoking, the risk for SUD appeared elevated for offspring with a father who smoked before pregnancy (HR = 2.4, 95% CI 1.3–4.5 for 1–9 cigarettes and HR = 2.3, 95% CI 1.6–3.3 for ≥10 cigarettes daily). However, paternal smoking before pregnancy did not increase the risk for mood or psychotic disorder for offspring at the age of 29–30. The association between paternal smoking before pregnancy and offspring anxiety disorder in young adulthood attenuated after adjustment for gender, family structure, parental psychiatric disorder and offspring illicit drug use at the age of 15–16 years. In contrast, the association between paternal smoking of ≥10 cigarettes daily before pregnancy and offspring SUD remained statistically significant after adjustments (HR = 1.8, 95% CI 1.03–3.1; Table 4).

**Table 4:** Adjusted Cox regression analyses for offspring psychiatric diagnoses with paternal smoking before pregnancy

	Any psychiatric disorder	Anxiety disorder	SUD**a**
Characteristics	HR (95%CI)	HR (95% CI)	HR (95% CI)
Paternal smoking before pregnancy			
<9 cigarettes	1.0 (0.7–1.3)	0.7 (0.4–1.2)	2.1 (0.8–5.2)
≥10 cigarettes	0.9 (0.8–1.1)	1.0 (0.8–1.2)	1.8 (1.04–3.1)
Maternal alcohol use during pregnancy (yes)	1.1 (0.9–1.4)	1.1 (0.8–1.5)	1.1 (0.6–2.1)
Female gender	1.5 (1.3–1.8)	1.9 (1.5–2.3)	0.5 (0.3–0.8)
Maternal education <12 years	1.1 (0.9–1.2)	1.2 (0.9–1.5)	0.8 (0.5–1.3)
Family structure			
One parent or other	1.2 (0.99–1.4)	1.3 (1.03–1.7)	1.8 (1.02–3.1)
Maternal psychiatric diagnosis (yes)	1.6 (1.3–1.8)	1.5 (1.2–1.9)	1.7 (1.01–2.9)
Paternal psychiatric diagnosis (yes)	1.6 (1.4–1.9)	1.5 (1.2–1.9)	3.2 (1.9–5.2)
Offspring daily smoking at the age of 15–16 years (yes)	1.1 (0.8–1.3)	1.1 (0.8–1.5)	2.7 (1.5–4.8)
Offspring intoxication frequency at the age of 15–16 years, past 30 days (yes)	1.2 (0.98–1.6)	1.1 (0.8–1.6)	1.5 (0.8–2.9)
Offspring other drug use at the age of 15–16 years (yes)	1.8 (1.4–2.3)	1.5 (1.1–2.2)	2.2 (1.2–4.1)
Paternal alcohol use during pregnancy (yes)			1.0 (0.5–1.8)
Maternal alcohol use at offspring age 15–16 years (yes)			0.9 (0.5–1.9)
Paternal alcohol use at offspring age 15–16 years (yes)			1.0 (0.6–1.8)
Interactions			SUD^a^
Paternal psychiatric diagnosis (no) × paternal smoking before pregnancy (yes)			3.0 (1.6–5.6)
Paternal psychiatric diagnosis (yes) × paternal smoking before pregnancy			
No smoking			5.1 (2.5–10.3)
Smoking			5.2 (2.6–10.4)

a Additional adjustment with paternal alcohol use during pregnancy and parental alcohol use at offspring age 15–16 years and interaction analyses were only made for offspring SUD. Interactions were adjusted with all confounding factors.

Noting the significant association between paternal daily smoking prior to pregnancy and offspring SUD, we then examined the prevalence of SUD among the 29- to 30-year-old offspring with a father who had or did not have any psychiatric diagnosis and who did or did not smoke daily pre-pregnancy (Supplementary data 3). The prevalence of SUD in the offspring with a father having no psychiatric diagnosis was 1.1% if they were non-smoking and 2.8% for those who smoked daily. In offspring with a father having any psychiatric diagnosis the prevalence of SUD was higher: 3.8% if the father did not smoke, and 5.5% if the father smoked every day. In a *post-hoc* interaction analysis, the risk for SUD was increased in offspring with a daily smoking father who did not have any psychiatric diagnosis even after adjusting for all the confounding factors (HR = 3.0, 95% CI 1.6–5.6; Table 4). The risk of SUD in offspring with a father who had any psychiatric diagnosis appeared to be elevated after adjustments, whether the father did not smoke or smoked daily (HR = 5.1, 95% CI 2.5–10.2 and HR = 5.2, 95% CI 2.6–10.4, respectively).

There were no statistically significant interactions between maternal smoking during pregnancy or paternal smoking before pregnancy and offspring intoxication frequency, daily smoking and other drug use at the age of 15–16 years when the outcome was either any psychiatric diagnosis, SUD, mood disorder or anxiety disorder. No statistically significant interactions were found between paternal smoking before pregnancy and any paternal psychiatric disorder in relation to offspring SUD.

Attrition from the sample studied in this work has been analyzed and presented previously.^21^ Fewer males (64% vs. 71%; χ^2^ test, *P* < 0.001), adolescents with parental psychiatric disorder (58% vs. 69%, *P* < 0.001) and subjects living in urban areas (66% vs. 71%, *P* < 0.001) participated in the follow-up study. Regarding psychiatric diagnoses, fewer of the diagnosed (65.1% vs. 74.2%, *P *<* *0.001) participants retained. Participants with missing data were excluded from the study. However, this did not have major impact on the distribution of covariates within outcomes (Supplementary data 4 and 5).

## Discussion

In this large birth cohort study using prospectively collected data over the life course, the associations between maternal smoking during pregnancy and psychiatric disorders in offspring were largely accounted for by adolescent smoking, alcohol and illicit substance use in parallel with parental psychiatric disorders. Thus, these factors seem to modulate the development of offspring psychiatric disorders in young adulthood, rather than maternal smoking during pregnancy or paternal smoking before pregnancy. SUD, however, did remain associated with heavier paternal smoking before pregnancy.

Maternal smoking during pregnancy has been found in previous studies to increase the risk of psychosis, mood, anxiety and alcohol use disorders and drug use in offspring.^4^^,^^5^^,^^22–24^ In these studies, the confounding factors related to time of birth of the child and not to later life stages of the offspring. Only in one birth cohort study, maternal minor psychiatric disorders at offspring age 11 years and adolescent’s mental health at the age of 15 years were considered.^22^ Contrarily, there are also results in which prenatal nicotine exposure was unrelated with offspring substance abuse and alcohol problems, depression and anxiety disorder.^25^ In our work, maternal smoking during pregnancy appeared to have a prospective association with adult offspring’s SUD, mood and anxiety disorders, but not with psychosis. Although, our results support the previous findings of maternal smoking during pregnancy increasing the risk for SUD, mood and anxiety disorder, the association attenuated after adjustment with parental psychiatric disorders and offspring’s own substance use. This suggests that psychiatric problems of the parents and offspring own drug use at the age of 15–16 years modulate the development of offspring’s later SUD, mood and anxiety disorders. Concerning SUD, both adolescent’s own use of drugs and daily smoking seem to modify the outcome in early adulthood.

Father’s smoking can cause DNA sequence mutations and epigenetic modifications in the sperm, which are then transmitted to the embryo and may potentially induce diseases in offspring.^14^ In a recent study, paternal smoking at conception was more common among offspring with ADHD, but the association attenuated after adjusting with paternal ADHD.^26^ In our work, paternal smoking before pregnancy did not increase the risk for mood disorder and psychosis. However, father’s daily smoking of ≥10 cigarettes appeared to increase the risk for adult offspring anxiety disorder. This association attenuated after adjusting with parental psychiatric disorders and offspring own tobacco smoking and illicit drug use at the age of 15–16 years, which indicates that these factors modulate the development of anxiety disorder in offspring. Contrarily, paternal smoking ≥10 cigarettes before pregnancy seemed to elevate the risk for adult offspring SUD and this association was stable regardless of the confounding factors used in all multivariable analyses and even after adjusting with father’s alcohol use during pregnancy and parental alcohol use at offspring age 15–16 years. However, also including maternal smoking into this model attenuated this association to non-significant. Nonetheless, although paternal daily smoking before pregnancy appears to elevate the risk for offspring SUD, the risk is even higher if the father has a psychiatric diagnosis regardless of paternal smoking status. Psychiatric problems in the father increasing the risk of offspring SUD is in line with previous studies.^27–29^ The association may be due to genetic and environmental factors that fathers pass on to their children. In summary, both paternal smoking before pregnancy and paternal psychiatric disorder may be early markers for offspring morbidity to SUD in early adulthood.

Adolescent smoking has been associated with SUD in early adulthood.^9^ Moreover, illicit drug use in teenage years has been linked to elevated risk of SUD and depression.^30^ The influence of drugs may work through disruption of brain development processes critical to the normal remodeling of brain during adolescence or to the development of unhelpful coping mechanisms.^31^ In addition, the risk of SUD, mood and anxiety disorders in the young adult offspring has been documented to be increased by parental psychiatric disorders.^32^ Dean et al.^32^ concluded that the risk was mediated by genetic and environmental factors. In this study, adolescent offspring tobacco smoking and other drug use and parental psychiatric diagnoses at offspring age 15–16 years were significant predictors of offspring psychiatric disorders.

### Strengths and limitations

Major strengths of this study are our use of a large population-based birth cohort with relatively small attrition, and comprehensive, high-quality register data of several psychiatric disorders. We used a prospective, longitudinal design and included information of offspring substance use in adolescence and parental psychiatric disorders. Limitations of this study include the use of self-report of parental smoking rather than cotinine or carbon monoxide breath analyzers to verify parental smoking behavior. No information about secondhand smoking exposure of the children was available. However, this is likely to under-estimate the prevalence of smoking exposures, and thus the strength of associations, so our findings may be considered conservative. Also, attrition at 15–16 years questionnaires may have affected the results. Furthermore, register data included only those study members with a psychiatric disorder diagnosed in national health care. However, data in this work cover actual diagnoses and more severe symptoms. Finally, 64.3% of the women who smoked before pregnancy had a smoking partner as a father of their offspring (data available on request). Therefore, assortative mating may have biased the results.

## Conclusions

When studying long-term psychiatric outcomes of maternal smoking during pregnancy, parental psychiatric disorders as well as adolescent substance use among offspring seem to modulate these associations suggesting that both early and more proximal factors may be influential. Therefore, information over the offspring life course is essential when considering the effect of maternal smoking during pregnancy and paternal smoking before pregnancy on their psychiatric disorders. Paternal smoking before pregnancy and paternal psychiatric diagnosis may act as modifiers in elevating the risk of SUD among offspring.

## Supplementary data

Supplementary data are available at *EURPUB* online.

## Supplementary Material

ckac004_Supplementary_Data
