# Supplementary material for: Parental smoking and young adult offspring psychosis, depression and anxiety disorders and substance use disorder
Source: Eur J Public Health. 2022 Jan 29;32(2):254–60. doi: 10.1093/eurpub/ckac004 (PMC9090280; doi:10.1093/eurpub/ckac004)
Supplement: ckac004_Supplementary_Data [file ckac004_supplementary_data.zip › ckac004-suppl_data/ejph-2021-04-om-0500-File002.docx]

**Supplement 1. Flowchart of the study**

Consent for use of participant data,

n = 7586 (80.4%)

Information on maternal smoking during pregnancy,

n = 8986 (95.3%)

and paternal smoking before pregnancy,

n = 8095 (85.8%)

Northern Finland Birth Cohort of 1986:

Children born alive,

n = 9432

Included in analyses

n = 7259 (77.0%)

Excluded from analyses if:

- Psychiatric diagnosis before age 16, n = 327 (3.5%)
