# Supplementary material for: Parental smoking and young adult offspring psychosis, depression and anxiety disorders and substance use disorder
Source: Eur J Public Health. 2022 Jan 29;32(2):254–60. doi: 10.1093/eurpub/ckac004 (PMC9090280; doi:10.1093/eurpub/ckac004)
Supplement: ckac004_Supplementary_Data [file ckac004_supplementary_data.zip › ckac004-suppl_data/ejph-2021-04-om-0500-File003.docx]

**Supplement 2. The ICD-10 codes for different categories of psychiatric disorders.**

| **Category** |  | **ICD-10 codes** | |  |
| --- | --- | --- | --- | --- |
| Any psychosis |  | F20-F25, F28, F29, F302, F312, F315, F323, F333 | | |
| Substance use disorders | | F101, F102, F111, F112, F121, F122, F131, F132, F141, F142, F151, F152, F161, F162, F171, F172, F181, F182, F191, F192 | | |
| Mood Disorders |  | F300-F301, F303-F311, F313-F314, F316-F319, F320-F322, F324-F332, F334-F339, F341, F3810 | | |
| Anxiety Disorders |  | F40-F44 |  |  |
