# Supplementary material for: Parental smoking and young adult offspring psychosis, depression and anxiety disorders and substance use disorder
Source: Eur J Public Health. 2022 Jan 29;32(2):254–60. doi: 10.1093/eurpub/ckac004 (PMC9090280; doi:10.1093/eurpub/ckac004)
Supplement: ckac004_Supplementary_Data [file ckac004_supplementary_data.zip › ckac004-suppl_data/ejph-2021-04-om-0500-File004.docx]

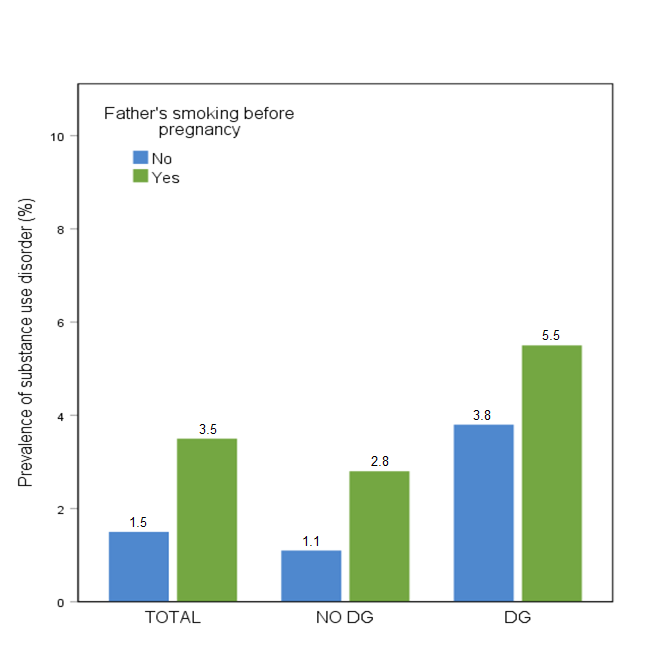


Supplement 3 Prevalence of offspring substance use disorder (%) by the age of 29 – 30 years as a function of paternal smoking before pregnancy and paternal psychiatric diagnose. NO DG = Offspring with a father who does not have a psychiatric diagnose and DG = Offspring with a father who has a psychiatric diagnose
