# Supplementary material for: Parental smoking and young adult offspring psychosis, depression and anxiety disorders and substance use disorder
Source: Eur J Public Health. 2022 Jan 29;32(2):254–60. doi: 10.1093/eurpub/ckac004 (PMC9090280; doi:10.1093/eurpub/ckac004)
Supplement: ckac004_Supplementary_Data [file ckac004_supplementary_data.zip › ckac004-suppl_data/ejph-2021-04-om-0500-File005.docx]

**Supplement 4. Characteristics of covariates vs. crude model and covariates vs. adjusted model in maternal smoking during pregnancy**

|  | **Crude model** | | | | | | | | | **Adjusted model** | | | | | | | | |
| --- | --- | --- | --- | --- | --- | --- | --- | --- | --- | --- | --- | --- | --- | --- | --- | --- | --- | --- |
|  | **Total (n=7259)** | **Any psychiatric disorder (n=1325)** | | **Anxiety disorder (n=672)** | | **Mood disorder (n=604)** | | **Substance use disorder (n=168)** | | **Total (n=4821)** | **Any psychiatric disorder (n=847)** | | **Anxiety disorder**  **(n=354)** | | **Mood disorder (n=329)** | | **Substance use disorder (n=59)** | |
|  | **n** | **n** | **%** | **n** | **%** | **n** | **%** | **n** | **%** | **n** | **n** | **%** | **n** | **%** | **n** | **%** | **n** | **%** |
| **Gender** |  |  |  |  |  |  |  |  |  |  |  |  |  |  |  |  |  |  |
| Male | 3593 | 521 | 14.5 | 240 | 6.7 | 207 | 5.8 | 105 | 2.9 | 2262 | 315 | 13.9 | 133 | 5.9 | 129 | 5.7 | 60 | 2.7 |
| Female | 3666 | 804 | 21.9 | 432 | 11.8 | 397 | 10.8 | 63 | 1.7 | 2559 | 532 | 20.8 | 276 | 10.8 | 249 | 9.7 | 33 | 1.3 |
| **Maternal smoking during pregnancy** |  |  |  |  |  |  |  |  |  |  |  |  |  |  |  |  |  |  |
| No | 5908 | 1040 | 17.6 | 511 | 8.6 | 475 | 8.0 | 120 | 2.0 | 4012 | 681 | 17.0 | 322 | 8.0 | 310 | 7.7 | 70 | 1.7 |
| 1-9 cigarettes a day | 659 | 138 | 20.9 | 78 | 11.8 | 54 | 8.2 | 22 | 3.3 | 402 | 82 | 20.4 | 43 | 10.7 | 29 | 7.2 | 11 | 2.7 |
| ≥10 cigarettes a day | 692 | 147 | 21.2 | 83 | 12.0 | 75 | 10.8 | 26 | 3.8 | 407 | 84 | 20.6 | 44 | 10.8 | 39 | 9.6 | 12 | 2.9 |
| **Paternal smoking before pregnancy** |  |  |  |  |  |  |  |  |  |  |  |  |  |  |  |  |  |  |
| No | 4083 | 713 | 17.5 | 362 | 8.9 | 335 | 8.2 | 62 | 1.5 | 2860 | 487 | 17.0 | 235 | 8.2 | 225 | 7.9 | 33 | 1.2 |
| 1-9 cigarettes a day | 333 | 60 | 18.0 | 25 | 7.5 | 24 | 7.2 | 12 | 3.6 | 214 | 37 | 17.3 | 14 | 6.5 | 14 | 6.5 | 7 | 3.3 |
| ≥10 cigarettes a day | 1915 | 381 | 19.9 | 201 | 10.5 | 170 | 8.9 | 67 | 3.5 | 1211 | 225 | 18.6 | 115 | 9.5 | 96 | 7.9 | 39 | 3.2 |
| **Maternal alcohol use during pregnancy** |  |  |  |  |  |  |  |  |  |  |  |  |  |  |  |  |  |  |
| No | 6267 | 1123 | 17.9 | 568 | 9.1 | 503 | 8.0 | 136 | 2.2 | 4201 | 728 | 17.3 | 353 | 8.4 | 319 | 7.6 | 76 | 1.8 |
| Yes | 905 | 186 | 20.6 | 94 | 10.4 | 92 | 10.2 | 28 | 3.1 | 620 | 119 | 19.2 | 56 | 9.0 | 59 | 9.5 | 17 | 2.7 |
| **Maternal education** |  |  |  |  |  |  |  |  |  |  |  |  |  |  |  |  |  |  |
| ≥12 years | 2019 | 337 | 16.7 | 158 | 7.8 | 154 | 7.6 | 32 | 1.6 | 1629 | 268 | 16.5 | 118 | 7.2 | 124 | 7.6 | 28 | 1.7 |
| <12 years | 4147 | 768 | 18.5 | 394 | 9.5 | 341 | 8.2 | 97 | 2.3 | 3192 | 579 | 18.1 | 291 | 9.1 | 254 | 8.0 | 65 | 2.0 |
| **Family type** |  |  |  |  |  |  |  |  |  |  |  |  |  |  |  |  |  |  |
| Both parents | 4851 | 787 | 16.2 | 383 | 7.9 | 342 | 7.1 | 76 | 1.6 | 3902 | 635 | 16.3 | 302 | 7.7 | 271 | 6.9 | 58 | 1.5 |
| One parent or other | 1347 | 326 | 24.2 | 178 | 13.2 | 162 | 12.0 | 55 | 4.1 | 919 | 212 | 23.1 | 107 | 11.6 | 107 | 11.6 | 35 | 3.8 |
| **Intoxication frequency^1,2^** |  |  |  |  |  |  |  |  |  |  |  |  |  |  |  |  |  |  |
| 0-2 | 5491 | 935 | 17.0 | 459 | 8.4 | 408 | 7.4 | 99 | 1.8 | 4411 | 746 | 16.9 | 363 | 8.2 | 327 | 7.4 | 71 | 1.6 |
| 3 or more | 579 | 138 | 23.8 | 69 | 11.9 | 74 | 12.8 | 33 | 5.7 | 410 | 101 | 24.6 | 46 | 11.2 | 51 | 12.4 | 22 | 5.4 |
| **Daily smoking^2^** |  |  |  |  |  |  |  |  |  |  |  |  |  |  |  |  |  |  |
| No | 5684 | 965 | 17.0 | 494 | 8.7 | 431 | 7.6 | 82 | 1.4 | 4280 | 712 | 16.6 | 344 | 8.0 | 307 | 7.2 | 57 | 1.3 |
| Yes | 842 | 226 | 26.8 | 116 | 13.8 | 117 | 13.9 | 60 | 7.1 | 541 | 135 | 25.0 | 65 | 12.0 | 71 | 13.1 | 36 | 6.7 |
| **Illicit drug use^2^** |  |  |  |  |  |  |  |  |  |  |  |  |  |  |  |  |  |  |
| No | 5732 | 949 | 16.6 | 476 | 8.3 | 411 | 7.2 | 99 | 1.7 | 4466 | 735 | 16.5 | 360 | 8.1 | 316 | 7.1 | 67 | 1.5 |
| Yes | 490 | 148 | 30.2 | 68 | 13.9 | 80 | 16.3 | 36 | 7.3 | 355 | 112 | 31.5 | 49 | 13.8 | 62 | 17.5 | 26 | 7.3 |
| **Maternal psychiatric disorder** |  |  |  |  |  |  |  |  |  |  |  |  |  |  |  |  |  |  |
| No | 5835 | 959 | 16.4 | 475 | 8.1 | 425 | 7.3 | 110 | 1.9 | 3937 | 623 | 15.8 | 300 | 7.6 | 263 | 6.7 | 61 | 1.5 |
| Yes | 1424 | 366 | 25.7 | 197 | 13.8 | 179 | 12.6 | 58 | 4.1 | 884 | 224 | 25.3 | 109 | 12.3 | 115 | 13.0 | 32 | 3.6 |
| **Paternal psychiatric disorder** |  |  |  |  |  |  |  |  |  |  |  |  |  |  |  |  |  |  |
| No | 5892 | 969 | 16.4 | 484 | 8.2 | 434 | 7.4 | 102 | 1.7 | 3974 | 621 | 15.6 | 299 | 7.5 | 275 | 6.9 | 52 | 1.3 |
| Yes | 1367 | 356 | 26.0 | 188 | 13.8 | 170 | 12.4 | 66 | 4.8 | 847 | 226 | 26.7 | 110 | 13.0 | 103 | 12.2 | 41 | 4.8 |

^1^ past 30 days.

^2^ at the age of 15-16 years.
