# Supplementary material for: Parental smoking and young adult offspring psychosis, depression and anxiety disorders and substance use disorder
Source: Eur J Public Health. 2022 Jan 29;32(2):254–60. doi: 10.1093/eurpub/ckac004 (PMC9090280; doi:10.1093/eurpub/ckac004)
Supplement: ckac004_Supplementary_Data [file ckac004_supplementary_data.zip › ckac004-suppl_data/ejph-2021-04-om-0500-File006.docx]

**Supplement 5. Characteristics of covariates vs. crude model and covariates vs. adjusted model in paternal smoking before pregnancy**

|  | **Crude model** | | | | | | | | **Adjusted model** | | | | | | |  |
| --- | --- | --- | --- | --- | --- | --- | --- | --- | --- | --- | --- | --- | --- | --- | --- | --- |
|  | **Total (n=6331)** | **Any psychiatric disorder (n=1154)** | | **Anxiety disorder (n=588)** | | **Substance use disorder (n=141)** | | **Total (n=4285)** | | **Any psychiatric disorder (n=749)** | | **Anxiety disorder**  **(n=364)** | | **Substance use disorder (n=79)** | | |
|  | **n** | **n** | **%** | **n** | **%** | **n** | **%** | **n** | | **n** | **%** | **n** | **%** | **n** | **%** | |
| **Gender** |  |  |  |  |  |  |  |  | |  |  |  |  |  |  | |
| Male | 3136 | 455 | 14.5 | 210 | 6.7 | 90 | 2.9 | 2009 | | 281 | 14.0 | 118 | 5.9 | 51 | 2.5 | |
| Female | 3195 | 699 | 21.9 | 378 | 11.8 | 51 | 1.6 | 2276 | | 468 | 20.6 | 246 | 10.8 | 28 | 1.2 | |
| **Maternal smoking during pregnancy** |  |  |  |  |  |  |  |  | |  |  |  |  |  |  | |
| No | 5158 | 910 | 17.6 | 450 | 8.7 | 100 | 1.9 | 3568 | | 602 | 16.9 | 287 | 8.0 | 57 | 1.6 | |
| 1-9 cigarettes a day | 567 | 121 | 21.3 | 69 | 12.2 | 20 | 3.5 | 354 | | 73 | 20.6 | 38 | 10.7 | 10 | 2.8 | |
| ≥10 cigarettes a day | 606 | 123 | 20.3 | 69 | 11.4 | 21 | 3.5 | 363 | | 74 | 20.4 | 39 | 10.7 | 12 | 3.3 | |
| **Paternal smoking before pregnancy** |  |  |  |  |  |  |  |  | |  |  |  |  |  |  | |
| No | 4083 | 713 | 17.5 | 362 | 8.9 | 62 | 1.5 | 2860 | | 487 | 17.0 | 235 | 8.2 | 33 | 1.2 | |
| 1-9 cigarettes a day | 333 | 60 | 18.0 | 25 | 7.5 | 12 | 3.6 | 214 | | 37 | 17.3 | 14 | 6.5 | 7 | 3.3 | |
| ≥10 cigarettes a day | 1915 | 381 | 19.9 | 201 | 10.5 | 67 | 3.5 | 1211 | | 225 | 18.6 | 115 | 9.5 | 39 | 3.2 | |
| **Maternal alcohol use during pregnancy** |  |  |  |  |  |  |  |  | |  |  |  |  |  |  | |
| No | 5491 | 977 | 17.8 | 497 | 9.1 | 115 | 2.1 | 3719 | | 635 | 17.1 | 310 | 8.3 | 63 | 1.7 | |
| Yes | 814 | 174 | 21.4 | 89 | 10.9 | 25 | 3.1 | 566 | | 114 | 20.1 | 54 | 9.5 | 16 | 2.8 | |
| **Maternal education** |  |  |  |  |  |  |  |  | |  |  |  |  |  |  | |
| ≥12 years | 1766 | 298 | 16.9 | 141 | 8.0 | 26 | 1.5 | 1444 | | 239 | 16.6 | 106 | 7.3 | 23 | 1.6 | |
| <12 years | 3659 | 679 | 18.6 | 349 | 9.5 | 84 | 2.3 | 2841 | | 510 | 18.0 | 258 | 9.1 | 56 | 2.0 | |
| **Family type** |  |  |  |  |  |  |  |  | |  |  |  |  |  |  | |
| Both parents | 4289 | 706 | 16.5 | 340 | 7.9 | 63 | 1.5 | 3495 | | 574 | 16.4 | 271 | 7.8 | 49 | 1.4 | |
| One parent or other | 1162 | 280 | 24.1 | 160 | 13.8 | 48 | 4.1 | 790 | | 175 | 22.2 | 93 | 11.8 | 30 | 3.8 | |
| **Intoxication frequency^1,2^** |  |  |  |  |  |  |  |  | |  |  |  |  |  |  | |
| 0-2 | 4809 | 817 | 17.0 | 399 | 8.3 | 83 | 1.7 | 3924 | | 660 | 16.8 | 324 | 8.3 | 60 | 1.5 | |
| 3 or more | 503 | 119 | 23.7 | 59 | 11.7 | 28 | 5.6 | 361 | | 89 | 24.7 | 40 | 11.1 | 19 | 5.3 | |
| **Daily smoking^2^** |  |  |  |  |  |  |  |  | |  |  |  |  |  |  | |
| No | 4981 | 842 | 16.9 | 431 | 8.7 | 66 | 1.3 | 3817 | | 636 | 16.7 | 309 | 8.1 | 47 | 1.2 | |
| Yes | 717 | 193 | 26.9 | 99 | 13.8 | 52 | 7.3 | 468 | | 113 | 24.1 | 55 | 11.8 | 32 | 6.8 | |
| **Illicit drug use^2^** |  |  |  |  |  |  |  |  | |  |  |  |  |  |  | |
| No | 5022 | 833 | 16.6 | 416 | 8.3 | 85 | 1.7 | 3972 | | 655 | 16.5 | 323 | 8.1 | 58 | 1.5 | |
| Yes | 418 | 123 | 29.4 | 56 | 13.4 | 29 | 6.9 | 313 | | 94 | 30.0 | 41 | 13.1 | 21 | 6.7 | |
| **Maternal psychiatric disorder** |  |  |  |  |  |  |  |  | |  |  |  |  |  |  | |
| No | 5109 | 840 | 16.4 | 419 | 8.2 | 93 | 1.8 | 3516 | | 558 | 15.9 | 271 | 7.7 | 53 | 1.5 | |
| Yes | 1222 | 314 | 25.7 | 169 | 13.8 | 48 | 3.9 | 769 | | 191 | 24.8 | 93 | 12.1 | 26 | 3.4 | |
| **Paternal psychiatric disorder** |  |  |  |  |  |  |  |  | |  |  |  |  |  |  | |
| No | 5141 | 854 | 16.6 | 432 | 8.4 | 86 | 1.7 | 3534 | | 554 | 15.7 | 271 | 7.7 | 45 | 1.3 | |
| Yes | 1190 | 300 | 25.2 | 156 | 13.1 | 55 | 4.6 | 751 | | 195 | 26.0 | 93 | 12.4 | 34 | 4.5 | |

^1^ past 30 days.

^2^ at the age of 15-16 years.
